# Ranitidine-Associated Sleep Disturbance: Case Report and Review of H2 Antihistamine-Related Central Nervous System Adverse Effects

**DOI:** 10.7759/cureus.2414

**Published:** 2018-04-03

**Authors:** Tyler Werbel, Philip R Cohen

**Affiliations:** 1 School of Medicine, University of California, San Diego, San Diego, USA; 2 Department of Dermatology, University of California, San Diego, San Diego, USA

**Keywords:** disturbance, dream, ranitidine, verruca, wart, zantac

## Abstract

Ranitidine is an H2 antihistamine used as an off-label therapy for recalcitrant verruca vulgaris. We describe a man who developed a sleep disturbance after initiating therapy with ranitidine and review similar adverse effects associated with other drugs in this class. The patient was a 40-year-old man with an eight-year history of a wart on his right plantar foot that was recalcitrant to several topical therapies. Adjunctive treatment with ranitidine 150 mg twice daily was initiated. He developed sleep disturbance with bizarre dreams and gastrointestinal symptoms. All symptoms resolved after discontinuation of the medication and recurred when he restarted the drug. PubMed was searched for the following terms: disturbance, dream, ranitidine, verruca, wart, and Zantac. The papers containing these terms and their references were reviewed. Sleep disturbance caused by ranitidine is an uncommon adverse event in patients receiving the drug. However, similar reactions have been observed with other H2 antihistamines such as cimetidine and famotidine. Clinicians should be aware that sleep disturbance secondary to ranitidine is a potential side effect of this medication.

## Introduction

Ranitidine is an H2 receptor antagonist that is typically used for the treatment of gastrointestinal conditions such as peptic ulcers or gastroesophageal reflux disease [1]; however, it is often used as off-label therapy for verruca vulgaris [2]. We describe a 40-year-old man who developed ranitidine-associated sleep disturbance when the medication was administered as adjunctive therapy for his recalcitrant plantar wart. Similar central nervous system adverse effects from other H2 antihistamines will be reviewed.

## Case presentation

A 40-year-old man presented for evaluation and treatment of a chronic verrucous lesion on the bottom of his right foot. The wart had been noted eight years earlier and had not responded to several topical therapies. His past medical history was significant for anaplastic lymphoma kinase (ALK)-negative anaplastic large cell lymphoma diagnosed three years ago. He was treated with chemotherapy, radiation, and an autologous bone marrow transplant. The most recent restaging scans two months ago had no evidence of relapse. The patient had no prior history of sleep disorders, including insomnia, narcolepsy, obstructive sleep apnea, and parasomnias.

Cutaneous examination of the medial aspect of his right plantar foot showed a verrucous plaque. Initial treatment included topical 40% salicylic acid pad applied daily and secured with duct tape. Once a week he used a pumice stone to manually pare the lesion.

Follow-up examination showed slight improvement. However, after continued similar management, the lesion persisted. Imiquimod five percent cream was prescribed to be applied on and around the wart each evening, and systemic adjunctive therapy was started with ranitidine 150 mg twice daily. Within three days, he developed gastrointestinal and central nervous system reactions. He described the gastrointestinal symptoms as gastroesophageal reflux disease-like with a constant full sensation in the middle of his chest.

The central nervous system symptoms consisted of difficulty falling asleep, disturbing dreams, and nighttime awakenings. In these dreams, he reported having a conversation with another person and being placed in situations that made him uncomfortable or threatened. However, he was unable to recall specific details. He explained that the dreams were bizarre, emotionally disturbing, and nonsensical. He also commented that the dreams caused him to experience a “different mental state” and would cause him to wake during the night.

After three weeks of twice daily ranitidine, he self-discontinued the medication; both the gastrointestinal and central nervous system symptoms promptly resolved. Three weeks later, he decided to take the ranitidine again; within one day, both symptoms recurred. Again, after stopping the ranitidine, all symptoms ceased and did not return.

## Discussion

H2 receptor blockers are a subclass of antihistamines that include cimetidine, ranitidine, famotidine, and nizatidine. They were marketed in 1977, 1983, 1986, and 1988 respectively. H2 antihistamines are effective in the treatment of peptic ulcer disease, gastroesophageal reflux disease, and hypersecretory conditions by indirectly reducing gastric acid secretion [1]. Cimetidine and ranitidine have also been used as off-label therapeutic interventions for warts [2].

Common side effects of H2 antihistamines include gastrointestinal disturbance (such as constipation, diarrhea, and nausea), headache, and skin rash. However, central nervous system adverse effects are uncommon [1, 3]. Similarly, H1 antihistamines have been reported to cause neuropsychiatric reactions, particularly in the elderly.

Due to the relatively low incidence, it is difficult to identify risk factors for central nervous system reactions to H2 antihistamines. However, review of the literature did not reveal sufficient evidence to suggest that age, H2 blocker dose, hepatic disease, immune status, renal disease, or concurrent use of other medications increased the likelihood of having such reactions. In 1991, Cantu et al. reviewed the central nervous system adverse effects associated with H2 antihistamines and found that cimetidine had been linked with central nervous system reactions more frequently than other drugs in its class. The neuropsychiatric adverse reactions of cimetidine included agitation, auditory and visual hallucinations, confusion, delirium, disorientation, psychosis, and somnolence [1].

Subsequently, nine patients with famotidine-associated mental status changes were described in three publications; clinical data from these individuals are summarized in Table 1 [4-6]. Albeit less common, there have been two patients who developed nizatidine-associated neuropsychiatric adverse effects. Following administration of nizatidine, a 93-year-old woman developed agitation, confusion, delirium, paranoid delusions, and visual and tactile hallucinations [7]; the other patient, a 16-year-old girl, experienced akathisia, bilateral upper extremity tremor, bradykinesia, and rigidity [8].

**Table 1:** Famotidine-associated central nervous system adverse effects ^a^Abbreviations: A, age (years); BID, twice daily; C, case; IV, intravenous; G, gender; Lat, latency (days); NS, not stated; PO, by mouth; PUD, peptic ulcer disease; Ref, reference; Res, resolution (days); UGIB, upper gastrointestinal bleed ^b^The initial dose was 20 mg IV BID for two days and was switched to 20 mg PO BID for one day. ^c^The initial dose was 20 mg BID for two days and was changed to ranitidine 150 mg BID for nine days. ^d^The latency was less than one day initially; when he restarted the drug, the latency was two days.

C^a^	A, G	Dose	Indication	Clinical features	Lat	Res	Ref
1	67, M	20 mg PO BID	Stress ulcer prophylaxis	Delirium, depression, lethargy	21	1	[4], C6
2	68, F	20 mg IV BID	Stress ulcer prophylaxis	Agitation, confusion, delirium, disorientation	4	2	[4], C3
3	68, M	^b^	PUD, UGIB	Hallucinations, irritability, mental status change, restlessness	2	1	[5], C1
4	70, M	20 mg BID	Stress ulcer prophylaxis	Delirium, disorientation, visual hallucinations	NS	2	[4], C1
5	72, F	20 mg IV BID	Stress ulcer prophylaxis	Agitation, confusion, delirium, talking to self	6	1	[4], C5
6	73, M	^c^	Stress ulcer prophylaxis	Agitation, confusion, delirium, disorientation, visual hallucinations	1	4	[4], C4
7	74, F	20 mg IV BID	Hematemesis	Confusion, disorientation	1	1	[5], C2
8	76, M	20 mg IV BID	Stress ulcer prophylaxis	Confusion, delirium	4	3	[4], C2
9	77, M	20 mg PO BID	Dyspepsia	Confusion, disorientation nightmares, restlessness	^d^	2	[6]

Das et al. demonstrated that – excluding headache – drowsiness and mental confusion were the most common neuropsychiatric side effects of ranitidine with incidences of 0.73 percent and 0.21 percent respectively [9]. Other central nervous system reactions include agitation, auditory and visual hallucinations, and delirium. Although less common, there have been reports of ranitidine-associated extrapyramidal symptoms, loss of color vision, mania, and late-onset depression [10].

To the best of our knowledge, sleep disturbance associated with H2 antihistamines has only been reported once in a 77-year-old man with dyspepsia. The patient’s medication was changed from ranitidine to famotidine due to a formulary change in his health maintenance organization; within four hours after his first evening dose, he experienced disturbing nightmares of a woman chasing him with an ax. Similar to our patient, symptoms resolved two days after self-discontinuation of the medication and returned after the patient decided to take famotidine again [6].

The pathogenesis of neuropsychiatric reactions associated with H2 antihistamines remains to be determined. However, cerebrospinal fluid analyses following oral ingestion of these drugs have demonstrated penetration of the blood-brain barrier by the H2 antihistamines. Therefore, interaction with H2 receptors in the central nervous system is an appealing explanation for the neuropsychiatric adverse effects; however, the role of histamine in the central nervous system is not completely established. Additionally, H2 blockers have demonstrated anticholinergic and gamma-aminobutyric acid-like properties that could contribute to central nervous system reactions [1].

In our patient, the diagnosis of ranitidine-associated sleep disturbance was confirmed when he decided to again challenge himself with the medication and subsequently developed the same adverse effect. It was further supported by the rapid amelioration of the symptoms after discontinuation of the drug. The sleep disturbance has not recurred since stopping the medication. The Naranjo scale was utilized to determine the probability that our patient’s sleep disturbance was due to ranitidine rather than a result of other factors. Our patient had a score of eight, indicating a probable adverse drug reaction.

## Conclusions

H2 blockers are currently available without prescription. Despite an excellent safety profile, adverse reactions may occur. Similar to our patient, central nervous system adverse events have been recorded. The pathogenesis of H2 antihistamine-associated neuropsychiatric adverse effects remains to be established. Our patient was able to confirm that ranitidine caused his sleep disturbance and dreams by again initiating treatment with the drug and promptly developing the identical central nervous system adverse effect. All of his symptoms resolved after he discontinued the medication. Therefore, albeit rare, clinicians should be aware of the potential central nervous system side effects that can occur in patients who receive ranitidine.
